# Selenium Protects against Zearalenone-Induced Oxidative Stress and Apoptosis in the Mouse Kidney by Inhibiting Endoplasmic Reticulum Stress

**DOI:** 10.1155/2020/6059058

**Published:** 2020-08-19

**Authors:** Yi Zhang, Bo Hu, Mingyang Wang, Jingjing Tong, Jianwen Pan, Nan Wang, Ping Gong, Miao Long

**Affiliations:** ^1^Key Laboratory of Zoonosis of Liaoning Province, College of Animal Science & Veterinary Medicine, Shenyang Agricultural University, Shenyang 110866, China; ^2^Institute of Animal Husbandry Quality Standards, Xinjiang Academy of Animal Science, Urumqi 830000, China

## Abstract

This study assessed the molecular mechanism of selenium (Se) protecting against kidney injury induced by zearalenone (ZEA) in mice. The experimental mice were divided into 4 groups including the control group, the Se group, the ZEA group, and the Se+ZEA group; ZEA and Se were administered orally for 28 days. The changes in renal biochemical index (BUN, UA, and CRE), biochemical change of kidney damage such as BUN, UA, and CRE, and oxidative damage such as MDA, T-SOD, and GSH-Px were investigated. Pathological sections and TUNEL staining were used to analyze renal pathological changes and cell apoptosis. qRT-PCR and Western blot were employed to detect the expression of genes and proteins which were related with endoplasmic reticulum stress. The results showed that ZEA increased the concentration of BUN, UA, and CRE and the content of MDA and decreased the activities of T-SOD and GSH-Px in the mouse kidneys. However, Se reversed above changes of the biochemical and antioxidant indexes of renal injury. Moreover, the results also showed that ZEA can increase the expression of Bax, caspase-12, caspase-3, Bip, CHOP, JNK protein, and mRNA and decrease the expression of Bcl-2 protein and mRNA. But Se reversed these proteins and genes related to endoplasmic reticulum stress and apoptosis. It can be concluded that Se protected against the kidney damage induced by ZEA. Se may protect the kidney from ZEA-induced apoptosis and oxidative stress by inhibiting ERS.

## 1. Introduction

Zearalenone (ZEA) is one of the most harmful mycotoxins produced primarily by Fusarium fungi [1]. The pollution of ZEA has become a worldwide public health problem [2] as ZEA can often be found in foods and feeds. China has set 60 *μ*g/kg as the maximum limit for ZEA in wheat and corn [3]. A provisional maximum tolerable daily intake (PMTDI) of ZEA is 0.5 *μ*g/kg [4]. Previous studies have shown that ZEA has severe reproductive toxicity, cytotoxicity, immunotoxicity, and carcinogenic effects on humans and livestock. In addition, ZEA and harmful substances, such as ammonia and chlorpyrifos, can also cause oxidative stress and apoptosis in the body (Fan et al. 2017, [5–8], Wanying et al. 2018, [9, 10]).

Current research focuses on the reproductive toxicity because ZEA has been shown to cause severe reproductive disorders in animals and hyperoestrogenic syndromes in humans as it has an oestrogenic effect ([11]; Zhang et al., [9, 10]). However, some results also have shown that ZEA not only causes serious reproductive toxicity but also has toxic effects on metabolic major organs such as the liver and kidney [12–14]. ZEA is excreted by the kidneys through the hepatoenteral circulation in animals (Rogowska et al. 2019). The accumulation of ZEA in the kidney induced glomerular atrophy and degeneration of renal tubular epithelial cells and induced proteinuria [14, 15]. By intraperitoneal injection of ZEA in female mice, Liang et al. (2015) have found that ZEA can accelerate renal cell apoptosis, increase serum creatinine and urea nitrogen levels, and cause renal oxidative stress in mice. The study from our group has also shown that ZEA can induce oxidative stress and apoptosis in the mouse kidney [15]. Therefore, it is very important to find an effective protective agent to protect the kidney from the damage caused by ZEA.

Selenium (Se) is an important trace element for the growth and development of humans and animals. In addition to antiaging and free radical scavenging properties, Se also has the beneficial effects in improving Alzheimer's disease, as an antitumor agent, enhancing immunity and regulating the reproductive system [16–19]. Zhou et al. (2009) have shown that Se can reduce cadmium-induced LLC-PK(1) cell apoptosis. Existing research shows that Se can prevent lead- and cadmium-induced kidney, liver, ovary, and testis cytotoxicity; oxidative stress; and endoplasmic reticulum stress, and research also shows that Se can also reduce lipopolysaccharide- (LPS-) induced myocarditis and nephropathy ([20–23], Wang et al., [24, 25]). In addition, a Se-deficient diet can lead to oxidative stress and hepatocyte apoptosis; dietary supplementation of Se reduces germ cell apoptosis in the testis [26, 27]. Se can also inhibit damage caused by mycotoxins. For example, dietary sodium selenite can significantly inhibit cell apoptosis caused by aflatoxin B1 [28]. Our previous studies also have shown that Se can alleviate the damage of ZEA on blood-testis barrier in male mice and inhibit ZEA-induced apoptosis and oxidative stress in mouse testis cells [29]. However, it is unclear whether Se can prevent ZEA from harming the kidneys and any protective underlying mechanism is yet to be elucidated.

The endoplasmic reticulum, an important organelle for eukaryotic cells, is used to synthesize proteins. It plays important roles in the synthesis, folding, and transport of proteins [30]. It is very sensitive to the changes in the intracellular environment that can regulate cell stress and calcium ion levels [31, 32]. Some exogenous substances, such as heavy metals and mycotoxins, induce endoplasmic reticulum stress and unfolded protein reactions that can result in apoptosis [24, 31, 33]. Studies have shown that some antioxidants such as resveratrol, procyanidin, and curcumin can inhibit apoptosis through inhibition of ERS [34–36]. Therefore, antioxidants can reduce the damage caused by foreign substances to cells by regulating endoplasmic reticulum stress.

It has been reported that oxidative stress is mainly induced by ZEA toxicity and some antioxidants can protect against ZEA toxicity. For example, proanthocyanidins can protect mouse small intestinal epithelial cells from ZEA-induced apoptosis through the ERS pathway (Miao et al. 2018); curcumin can inhibit ZEA-induced apoptosis and oxidative stress in Leydig cells [37].

Crocin and quercetin can inhibit ZEA-induced apoptosis in HCT116 and HEK293 cells by reducing ERS [38]. However, the results of Se in antioxidant capacity aspect do not show consistent results, and data on the mechanism of Se protection against ZEA-induced specific toxic injury have not been widely reported.

So in this study, whether Se can inhibit ZEA-induced oxidative stress and apoptosis in the mouse kidney via the inhibition of ERS was studied. The results can give us a theoretical basis for the use of Se to prevent toxicity of ZEA.

## 2. Materials and Methods

### 2.1. Animals

The 40 male Kunming mice were obtained from China Medical University, which weighed 20 ± 2 g and aged 4 weeks. They were bred in a restricted-access room, the room temperature was set from 22°C to 24°C and the humidity was set from 45% to 60%, and light and darkness were alternated every 12 h. The mice could get water and the controlled diet ad libitum. The animal experiment was carried out in accordance with the good principles of experimental animal care and European Communities Council Directive (86/609/EEC) and EU Directive 2010/63/EU and approved by the experimental animal care and use ethics committee of Shenyang Agricultural University, China (Permit No. 264SYXK<Liao>2011-0001, September 2018).

### 2.2. Experimental Design and Treatment

ZEA was purchased from Sigma (St. Louis, MO, USA), which was prepared with dimethyl sulfoxide (DMSO). And the storage concentration of ZEA was 100 mg/mL. The working solution of ZEA was diluted with normal saline. The working concentration of ZEA was set to 1 mg/mL: at this concentration, the DMSO content was 0.5%. Se yeast was obtained from the Angel Yeast Co. Ltd. (Wuhan, China; with a Se content of 2 g/kg).

The ZEA group (ZEA: 40 mg/kg dose of ZEA b.wt.), which was based on other previous experiments [29, 39] and Boeira and Zourgui (Boeira et al., 2014; Zourgui et al., 2008); Se group (0.4 mg/kg Se b.wt.) which was based on other previous experiments [29], Se+ZEA group (0.4 mg/kg Se b.wt.; 40 mg/kg dose of ZEA b.wt.). At 9:00 every day, the mice were intragastrically administered with Se solution, followed by ZEA solution after 40 min. The experiment lasted for 28 days.

At the end of the experiment, the mouse blood samples were obtained after euthanasia via anesthesia. The serum was obtained after the blood samples were separated at 3000 rpm for 20 min and stored at -20°C for later use. A portion of kidney tissue was homogenized in 1 mL of 10 mM Tris-HCl, pH 7.4 at 4°C. The other kidney samples were put into the cryopreservation tube and stored at -80°C for subsequent test.

### 2.3. Biochemical Assays

The concentration of blood urea nitrogen (BUN), urate (UA), and creatinine (CRE) in serum was determined according to Wang et al. [39]. The suspension of the kidney homogenate was collected and assayed for the activities of T-SOD (total superoxide dismutase) and GSH-Px (glutathione peroxidase) and the content of MDA (malondialdehyde). Each measurement was based on the manufacturer's instructions (Nanjing Jiancheng Institute of Bioengineering, China).

### 2.4. Organ Indexes and HE Staining

All mice were weighed after the last treatment. The organ indexes were obtained from the percentage of kidney wet weight to the total mouse body weight. HE staining was used to examine the pathological changes (three kidney tissues randomly selected from each group were examined).

### 2.5. TUNEL Analysis of Apoptosis

The apoptosis was measured by using TUNEL analysis. The operation methods and procedures are according to the manufacturer's instructions of the commercial kit (Roche, Beijing, China). The cells with green fluorescence are defined as TUNEL-positive cells. Aperio ImageScope software was used to count the number of TUNEL-positive cells and it was counted as a percentage of positive cells. Three independent statistics for the experiment were done, each time three technical repeats.

### 2.6. qRT-PCR

The TRIzol method (TaKaRa, Japan) was used to extract the total RNA of the kidney sample. The microvolume spectrometer at an absorbance ratio of 260/280 nm was used to measure the purity and concentration of RNA. The HiScript II QRT SuperMix for qPCR (+gDNA wiper) kit (Vazyme, Nanjing, China) was used to reverse the total RNA to cDNA.

An ABI 7500 real-time PCR system and the ChamQ Universal SYBR qPCR Master Mix kit were used to conduct real-time PCR. The primers were listed in Table 1. The procedure and condition of real-time PCR were conducted based on our previous study [29]. The relative changes in mRNA were calculated using the 2^−*ΔΔ*Ct^ method. Primers are designed and synthesized by Beijing Sangon Biotech Co., Ltd.

### 2.7. Western Blot Analysis

A protein extraction kit (Beyotime, Wuhan, China) was used to extract the protein of kidney tissue. The Pierce BCA Protein Assay Kit (Thermo Fisher) was used to measure total protein content. The protein samples were separated using a 10% SDS-PAGE, and then, the proteins were transferred to nitrocellulose by using a Trans-Blot machine (Thermo Fisher). The membrane was blocked for 2 hours at 25°C with TBST (Sangon Biotech, Shanghai, China) buffer diluted with BSA to 5% blocking solution. Membranes were added with the first antibodies then incubated overnight at 4°C: the first antibodies were *β*-actin, GRP78/Bip, JNK, p-JNK, CHOP, Bax, Bcl-2, caspase-12, and caspase-3. *β*-Actin was set as internal reference. The membrane was washed five times (each time for 10 min) with TBST buffer, then incubated with the secondary antibody at 37°C for 1 h and then washed five times (each time for 10 min). The Luminescent Image Analyzer (Ncmbio, Suzhou, China) was used to visualize and analyze the expressed protein.

### 2.8. Statistical Analysis

SPSS 19.0 software was used for the statistical test, and the results were expressed as mean ± standard deviation. Two-tailed Student's *t*-tests were used to analyze the difference between two groups, and significant differences among multiple groups were evaluated by using one-way analysis of variance (ANOVA) and LSD methods. Differences of *P* < 0.05 were considered significant.

## 3. Results

### 3.1. Organ Indices of Kidney and Blood Biochemistry

**Figure 1:**
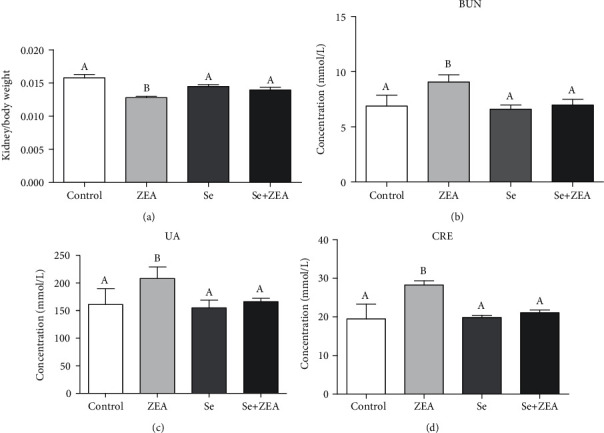
(a) Changes in the ratio of kidney weight to body weight in mice. (b) The content of BUN in the blood of mice. (c) The content of UA in the blood of mice. (d) The content of CRE in the blood of mice. The column diagrams marked by different letters were significant to each other (*P* < 0.05). Values were mean ± SD (*n* = 3).

**Figure 2:**
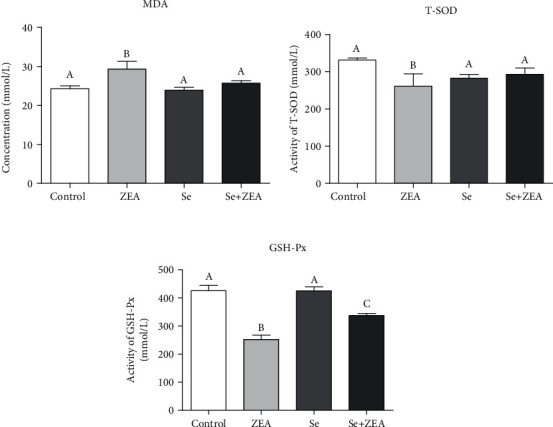
Oxidation and antioxidation parameters of renal tissues of mice in each group were detected by an oxidation kit. The column diagrams marked by different letters were significant to each other (*P* < 0.05). Values were mean ± SD (*n* = 3).

As shown in Figure 1(a), compared with the control group, the kidney index was decreased in the ZEA group (*P* < 0.05). However, compared with that of the ZEA group, the index of the Se+ZEA group was increased (*P* < 0.05). As shown in Figure 2, compared with the control group, the concentration of BUN, UA, and CRE in serum was increased in the ZEA group (*P* < 0.05). Compared with those in the control group, the three biomarkers in the Se group and the Se+ZEA group were not significantly different. Meanwhile, the three biomarkers in serum were significantly decreased in the Se+ZEA group, when compared to those in the ZEA group (*P* < 0.05).

### 3.2. Antioxidative Indexes in Renal Tissue

As shown in Figure 2, compared to the control group, the content of MDA was significantly increased (*P* < 0.05), while the activities of GSH-Px and T-SOD in the ZEA group were decreased (*P* < 0.05). Meanwhile, compared to the ZEA group, the activities of GSH-Px and T-SOD in the Se group and the Se+ZEA group were increased (*P* < 0.05), and the content of MDA was significantly decreased (*P* < 0.05). Therefore, oral administration of the Se significantly inhibited the increase in MDA content (*P* < 0.05) caused by ZEA, and it reversed the decreases in T-SOD and GSH-Px activities caused by ZEA.

### 3.3. Histopathological Changes in the Kidney

**Figure 3:**
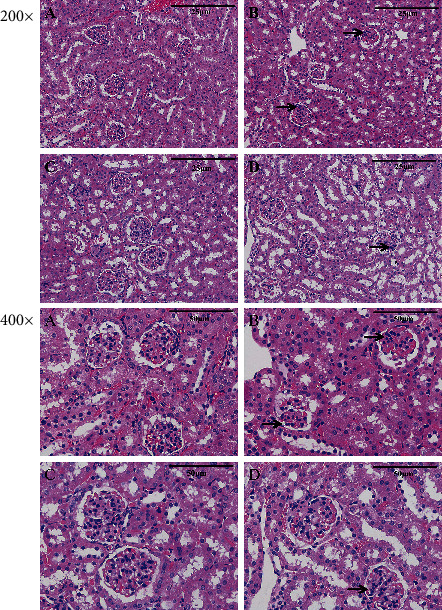
Detection of pathological changes in renal tissue by tissue section HE staining. Images were taken at a magnification of ×200 and ×400: (a) control group, (b) ZEA group, (c) Se group, and (d) Se+ZEA group. The arrows “→” indicate a pathological injury in the kidney, such as glomerulus lobulation and glomerulus atrophy.

The results showed that ZEA caused lobulation and atrophy of the glomerulus in the murine kidney (Figure 3). Furthermore, kidney sections from the Se and Se+ZEA groups revealed minor pathomorphological changes (Figure 3).

### 3.4. Effect of Se on Apoptosis of Kidney Cells in Mice Treated with ZEA

**Figure 4:**
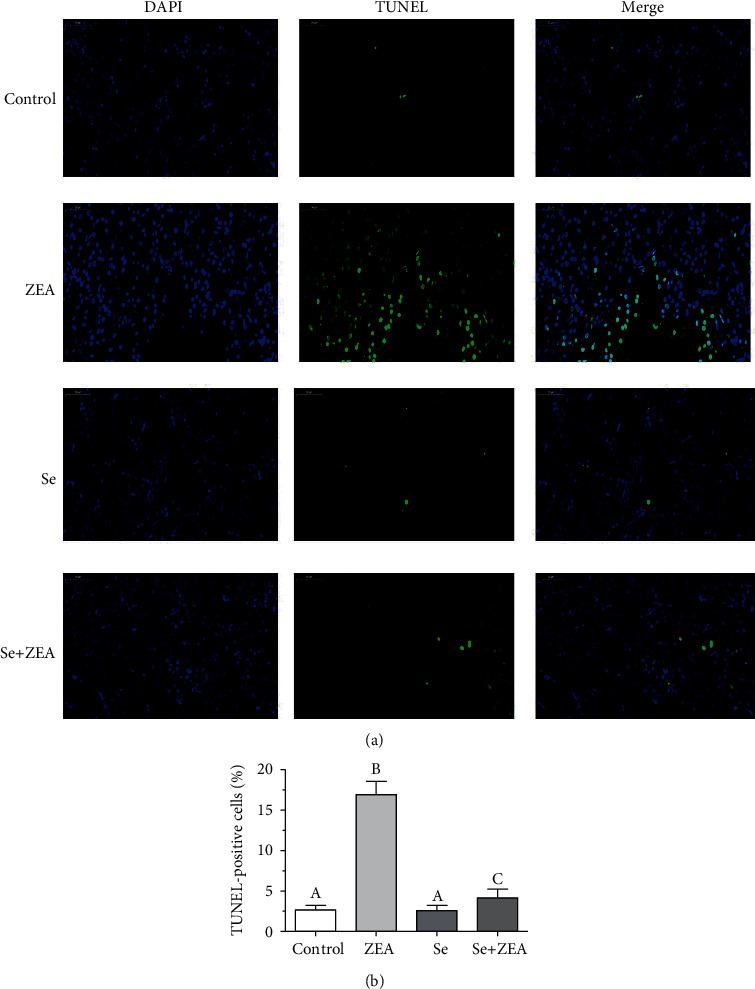
(a) Effects of Se on ZEA-induced apoptosis of renal cells. TUNEL-stained kidney section (magnification, ×50), with green granules indicating the positive cell. DAPI was used for nuclear staining. (b) The percentage of TUNEL-positive cells in each group. The column diagrams marked by different letters were significant to each other. Values were mean ± SD (*n* = 3).

**Figure 5:**
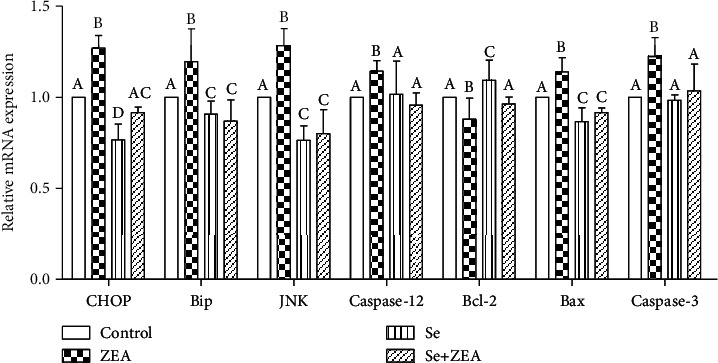
Effects of Se on the expression levels of genes involved in the endoplasmic reticulum stress signaling pathway that was induced by ZEA in the kidneys of mice. The column diagrams marked by different letters were significant to each other. Values were mean ± SD (*n* = 3).

As shown in Figure 4(a), we used the TUNEL method to analyze the effect of Se on renal cell apoptosis in ZEA-treated mice. Fragmented nuclei were typical characteristics of apoptosis; we performed DAPI staining and a TUNEL assay to detect alterations in the nuclei. As shown in Figure 5(b), the number of TUNEL-positive cells in the ZEA group increased significantly (*P* < 0.05) compared with that in the control group. Meanwhile, the number of positive cells in the Se+ZEA group decreased significantly (*P* < 0.05) compared with that in the ZEA group. The TUNEL-positive cells in the Se group were not significant compared with those in the control group.

### 3.5. Gene and Protein Expression

**Figure 6:**
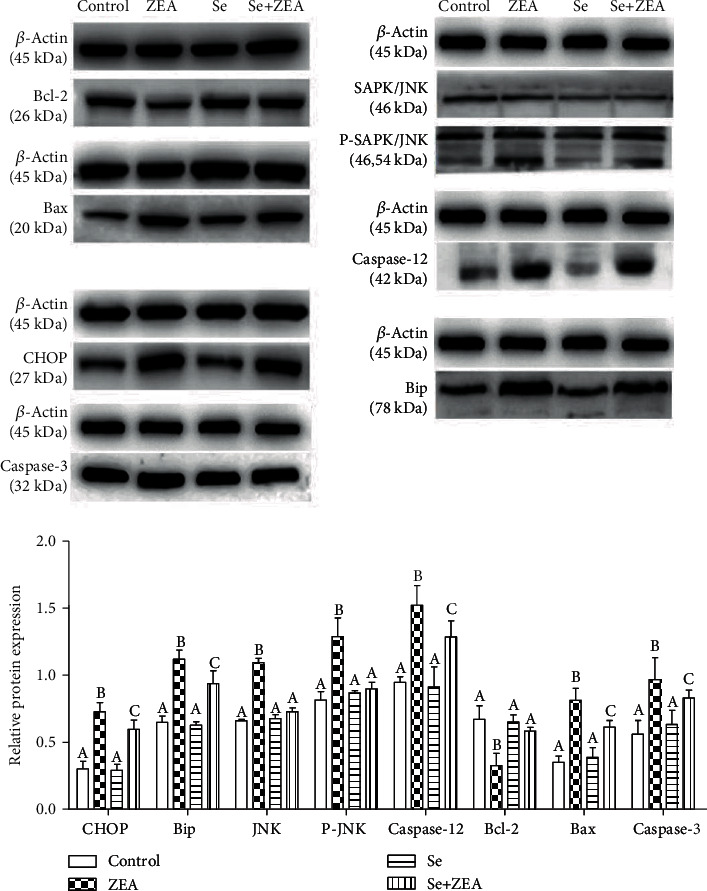
Effects of Se on the expression levels of proteins involved in the endoplasmic reticulum stress signaling pathway that was induced by ZEA in the kidneys of mice. The column diagrams marked by different letters were significant to each other. Values were mean ± SD (*n* = 3).

As shown in Figures 5 and 6, when compared with the control group, the mRNA and protein expression of Bip, JNK, p-JNK, caspase-12, caspase-3, Bax, and CHOP was significantly increased (*P* < 0.05) while the mRNA and protein expression of Bcl-2 significantly decreased (*P* < 0.05) in the ZEA group. In addition, when compared with the ZEA group, the expressions of mRNA and protein of Bip, JNK, p-JNK, caspase-12, caspase-3, Bax, and CHOP in the Se group and the Se+ZEA group were significantly decreased (*P* < 0.05), while these expressions of Bcl-2 were significantly increased. It is worth noting that in the Western blot results, compared to the Se group, the expressions of CHOP, Bip, caspase-12, caspase-3, and Bax were significantly increased in the Se+ZEA group.

## 4. Discussion

The kidney is an important metabolic organ in the body involved in the metabolism of drugs and toxins. However, whether ZEA can cause kidney damage and its mechanism are not clear. Therefore, we consider it necessary to study the kidney damage caused by ZEA and to find effective protective drugs. According to this study, ZEA can also cause kidney damage and can cause renal tissue apoptosis through endoplasmic reticulum stress.

Previous studies have proved that Se has many important biological functions. However, whether Se can antagonize the toxicity of mycotoxin and its mechanism of detoxification is not clear. If it can be proved that Se can alleviate the toxicity and mechanism of mycotoxin, it will be of great significance to prevent and control mycotoxin poisoning in animals. So our research further explores the effects and mechanisms of Se in antagonizing mycotoxins.

In this study, the dose of ZEA (40 mg/kg) is used in studying ZEA toxicity in mice. The choice of the doses of ZEA was based on preliminary experiments in our laboratory [15, 29] and other researchers' results (Boeira et al., 2014; Zourgui et al., 2008). These previous studies have showed that ZEA (40 mg/kg) given to mice could cause oxidative stress in the liver, kidney, and testis. And our results proved again that at this dose, ZEA can cause kidney damage.

As we all know the biochemical markers, BUN, UA, and CRE, are commonly used to assess kidney damage [15]. Our results showed that the levels of BUN, UA, and CRE in the kidney tissue of the ZEA group were significantly different from those of the control group after 28 days of administration of 40 mg/kg ZEA (Figure 1). In this study, MDA was used as an indicator of oxidative damage to the kidney, and the activities of GSH-Px and T-SOD in the kidney were used as indicators of antioxidants. Excess hazardous substance ammonia caused chicken oxidative stress, decreased GSH-Px, and increased MDA in chickens [6, 40], and chlorpyrifos caused common carp oxidative stress, decreased SOD, and increased MDA [41]. The results showed that ZEA caused damage to the mouse kidneys and produced oxidative stress as it could decrease the activities of GSH-Px and T-SOD and increase the content of MDA. In addition, the results of tissue sections and TUNEL staining showed ZEA caused damage to the kidneys of mice along with apoptosis in the kidney (Figures 3 and 4).

As a trace element, Se is an antioxidant that can eliminate oxidative free radicals generated by external toxicants, reduce the damage of oxidative stress on humans and animals, and improve the antioxidant capacity and immune function of humans and animals [42]. Studies have shown that Se deficiency is associated with higher mortality at birth and growth kinetics in rats, and at the same time, the liver of rats also developed slowly, the activity of GPX and CAT was decreased, and the activity of SOD was increased, but dietary Se improved all of these indices [43]. By contrast, when Se intake was excessive, the biochemical indicators of the metabolic organs such as the liver and kidneys of the body are affected which can be manifested as Se poisoning [44]. It was suggested that the intake of Se in adult males and females during pregnancy should be 55 *μ*g/day and 55-60 *μ*g/day, respectively [16, 44]. Therefore, the role of Se is highly dose dependent. Our results showed that there were no significant differences in kidney biochemical parameters such as kidney index and the content of BUN, UA, and CRE between the Se alone group and the control group. The results indicate that the dose of Se used in this study was safe and is consistent with our previous findings [29]. The renal antioxidant indexes of the Se+ZEA group were significantly changed than those of the ZEA group, indicating an improvement in the renal index. Generally, cotreatment with Se+ZEA improved the level of renal biochemical markers such as BUN, UA, and CRE and reversed oxidative indicators (Figures 1 and 2). The results of the renal histopathology and TUNEL staining indicated that Se can improve kidney damage caused by ZEA and inhibit renal cell apoptosis caused by ZEA (Figures 3 and 4) and indicated that Se is a good protective agent against ZEA-induced damage in the kidney.

In the endoplasmic reticulum stress pathway, GRP78 (Bip) protein is separated from transmembrane signaling protein, which leads to the activation of apoptotic proteins (CHOP, JNK, and caspase-12) and promotes apoptosis [45]. Our results indicate that ZEA promotes the expression of endoplasmic RNA and protein. In contrast, oral Se can significantly downregulate the expression of Bip protein and suggests that Bip protein may be a target of Se in renal tissue. Generally speaking, when ZEA induces endoplasmic reticulum stress in renal cells, the expression of CHOP protein increases resulting in the inhibition of the expression of downstream protein Bcl-2, which promotes apoptosis [46, 47]. However, when cells are stimulated by the outside world, the protein JNK in the cytoplasm is transformed into p-JNK and phosphorylated and enters the nucleus to promote the expression of apoptotic protein Bax [48, 49]. Caspase-12 has no activity when the cells are in normal state. However, procaspase-12 converts to caspase-12 when stimulated and activates downstream caspase-3 and caspase-9 to sense apoptosis [50]. Our results showed that the expressions of CHOP, Bip, JNK, caspase-12, Bax, Bcl-2, and caspase-3 in the Se+ZEA group were significantly improved compared to those in the ZEA group (Figures 5 and 6). This suggests that Se acts as a protective agent against ZEA-induced injury by acting on the endoplasmic reticulum stress pathway in the kidney.

## 5. Conclusions

In conclusion, ZEA-induced renal tissue damage is associated with ZEA-induced oxidative damage and apoptosis. Se can protect against kidney damage induced by ZEA by inhibiting ER stress. This study will provide Se with a molecular theoretical basis for preventing ZEA-induced kidney damage.

## Figures and Tables

**Table 1:** Primers for qRT-PCR analysis.

Gene	Accession no.	Primer sequences (5′-3′)	Product size (bp)
*β*-Actin	BC_138614.1	Forward: CTGTCCCTGTATGCCTCTG Reverse: TTGATGTCACGCACGATT	221 bp

GRP78/Bip	NM_001163434.1	Forward: CGCTGGGCATCATTGAAGTAA Reverse: GAGGTGGGCAAACCAAGACAT	145 bp

JNK	NM_001310452.1	Forward: TCCTCCAAATCCATTACCTCC Reverse: CTCCAGCACCCATACATCAAC	149 bp

Caspase-12	NM_009808.4	Forward: CTCAATAGTGGGCATCTGGGT Reverse: GAAGGTAGGCAAGACTGGTTC	151 bp

Bcl-2	NM_009741.5	Forward: CTCAGGCTGGAAGGAGAAGAT Reverse: AAGCTGTCACAGAGGGGCTAC	156 bp

Bax	NM_007527.3	Forward: GCAAAGTAGAAGAGGGCAACC Reverse: ACTGGACAGCAATATGGAGCT	156 bp

CHOP	NM_001290183.1	Forward: TTCTCCTTCATGCGTTGCTTC Reverse: AAAACCTTCACTACTCTTGACCCTG	218 bp

Caspase-3	NM_001284409.1	Forward: GAAACTCTTCATCATTCAGGCC Reverse: GCGAGTGAGAATGTGCATAAAT	250 bp

## Data Availability

The data used to support the findings of this study are included within the article.

## Abbreviations

ZEA: Zearalenone
ERS: Endoplasmic reticulum stress
BUN: Blood urea nitrogen
UA: Urate
CRE: Creatinine
MDA: Malondialdehyde
T-SOD: Total superoxide dismutase
GSH-Px: Glutathione peroxidase
Bip: Heavy chain binding protein
CHOP: C/EBP homologous protein
JNK: c-Jun N-terminal kinase
Caspase-12: Cysteinyl aspartate specific proteinase-12
Caspase-3: Cysteinyl aspartate specific proteinase-3
Bax: B-cell lymphoma-2
Bcl-2: BCL2-associated X.

## References

[1] Vejdovszky K., Hahn K., Braun D., Warth B., Marko D. (2017). Synergistic estrogenic effects of Fusarium and Alternaria mycotoxins in vitro. *Archives of Toxicology*.

[2] Simmonds M. S. J. (2004). IARC Monographs on the Evaluation of Carcinogenic Risks to Humans. Vol. 82, Some Traditional Herbal Medicines, Some Mycotoxins, Naphthalene and Styrene: World Health Organisation, IARC Press, Lyon France, 2002, 590 pp., ISBN 92 832 1282 7, US$40. *Phytochemistry*.

[3] Kong W.‐. J., Shen H.‐. H., Zhang X.‐. F. (2013). Analysis of zearalenone and*α*-zearalenol in 100 foods and medicinal plants determined by HPLC-FLD and positive confirmation by LC-MS-MS. *Journal of the science of food and agriculture*.

[4] Zinedine A., Soriano J. M., Molto J. C., Manes J. (2007). Review on the toxicity, occurrence, metabolism, detoxification, regulations and intake of zearalenone: an oestrogenic mycotoxin. *Food and Chemical Toxicology*.

[5] Kowalska K., Habrowska-Górczyńska D. E., Piastowska-Ciesielska A. W. (2016). Zearalenone as an endocrine disruptor in humans. *Environmental Toxicology and Pharmacology*.

[6] Shah S. W. A., Chen J., Han Q., Xu Y., Ishfaq M., Teng X. (2020). Ammonia inhalation impaired immune function and mitochondrial integrity in the broilers bursa of fabricius: implication of oxidative stress and apoptosis. *Ecotoxicology and Environmental Safety*.

[7] Shi B., Su Y., Chang S., Sun Y., Meng X., Shan A. (2017). Vitamin C protects piglet liver against zearalenone-induced oxidative stress by modulating expression of nuclear receptors PXR and CAR and their target genes. *Food & Function*.

[8] Wang M., Wang N., Tong J., Pan J., Long M., Li P. (2018). Transcriptome analysis to identify the Ras and Rap1 signal pathway genes involved in the response of TM3 Leydig cells exposed to zearalenone. *Environmental Science and Pollution Research*.

[9] Zhang K., Tan X., Li Y. (2018). Transcriptional profiling analysis of zearalenone-induced inhibition proliferation on mouse thymic epithelial cell line 1. *Ecotoxicology and Environmental Safety*.

[10] Zhang G. L., Feng Y. L., Song J. L. (2018). Zearalenone: a mycotoxin with different toxic effect in domestic and laboratory animals’ granulosa cells. *Frontiers in Genetics*.

[11] Rai A., Das M., Tripathi A. (2019). Occurrence and toxicity of a fusarium mycotoxin, zearalenone. *Critical Reviews in Food Science and Nutrition*.

[12] Gao X., Xiao Z. H., Liu M. (2018). Dietary silymarin supplementation alleviates zearalenone-induced hepatotoxicity and reproductive toxicity in rats. *The Journal of Nutrition*.

[13] Jia Z., Liu M., Qu Z., Zhang Y., Yin S., Shan A. (2014). Toxic effects of zearalenone on oxidative stress, inflammatory cytokines, biochemical and pathological changes induced by this toxin in the kidney of pregnant rats. *Environmental Toxicology and Pharmacology*.

[14] Wang L., Shi X., Zheng S., Xu S. (2020). Selenium deficiency exacerbates LPS-induced necroptosis by regulating miR-16-5p targeting PI3K in chicken tracheal tissue. *Metallomics*.

[15] Wang N., Li P., Pan J. (2018). *Bacillus velezensis* A2 fermentation exerts a protective effect on renal injury induced by zearalenone in mice. *Scientific Reports*.

[16] Qazi I. H., Angel C., Yang H. (2018). Selenium, selenoproteins, and female reproduction: a review. *Molecules*.

[17] Tan H. W., Mo H. Y., Lau A. T. Y., Xu Y. M. (2019). Selenium species: current status and potentials in cancer prevention and therapy. *International Journal of Molecular Sciences*.

[18] Tsuji P. A., Carlson B. A., Anderson C. B., Seifried H. E., Hatfield D. L., Howard M. T. (2015). Dietary selenium levels affect selenoprotein expression and support the interferon-*γ* and IL-6 immune response pathways in mice. *Nutrients*.

[19] Zheng R., Zhang Z. H., Chen C. (2017). Selenomethionine promoted hippocampal neurogenesis via the PI3K-Akt-GSK3*β*-Wnt pathway in a mouse model of Alzheimer’s disease. *Biochemical and Biophysical Research Communications*.

[20] Chen X., Zhu Y. H., Cheng X. Y., Zhang Z. W., Xu S. W. (2012). The protection of selenium against cadmium-induced cytotoxicity via the heat shock protein pathway in chicken splenic lymphocytes. *Molecules*.

[21] Liu J., Wang S., Zhang Q., Li X., Xu S. (2020). Selenomethionine alleviates LPS-induced chicken myocardial inflammation by regulating the miR-128-3p-p38 MAPK axis and oxidative stress. *Metallomics*.

[22] Liu L., Yang B., Cheng Y., Lin H. (2015). Ameliorative effects of selenium on cadmium-induced oxidative stress and endoplasmic reticulum stress in the chicken kidney. *Biological Trace Element Research*.

[23] Wan N., Xu Z., Liu T., Min Y., Li S. (2018). Ameliorative effects of selenium on cadmium-induced injury in the chicken ovary: mechanisms of oxidative stress and endoplasmic reticulum stress in cadmium-induced apoptosis. *Biological Trace Element Research*.

[24] Wang X., An Y., Jiao W. (2018). Selenium protects against lead-induced apoptosis via endoplasmic reticulum stress in chicken kidneys. *Biological Trace Element Research*.

[25] Zhang C., Lin J., Ge J. (2017). Selenium triggers Nrf2-mediated protection against cadmium-induced chicken hepatocyte autophagy and apoptosis. *Toxicology in vitro : an international journal published in association with BIBRA*.

[26] Song R., Yao X., Shi L., Ren Y., Zhao H. (2015). Effects of dietary selenium on apoptosis of germ cells in the testis during spermatogenesis in roosters. *Theriogenology*.

[27] Yao L., Du Q., Yao H., Chen X., Zhang Z., Xu S. (2015). Roles of oxidative stress and endoplasmic reticulum stress in selenium deficiency-induced apoptosis in chicken liver. *Biometals : an international journal on the role of metal ions in biology, biochemistry, and medicine*.

[28] Yu Z., Wang F., Liang N. (2015). Effect of selenium supplementation on apoptosis and cell cycle blockage of renal cells in broilers fed a diet containing aflatoxin B1. *Biological Trace Element Research*.

[29] Long M., Yang S., Wang Y. (2016). The protective effect of selenium on chronic zearalenone-induced reproductive system damage in male mice. *Molecules*.

[30] Barlowe C. K., Miller E. A. (2013). Secretory protein biogenesis and traffic in the early secretory pathway. *Genetics*.

[31] Ben Salem I., Boussabbeh M., Prola A. (2016). Crocin protects human embryonic kidney cells (HEK293) from *α*- and *β*-zearalenol-induced ER stress and apoptosis. *Environmental Science and Pollution Research International*.

[32] Kanemoto S., Nitani R., Murakami T. (2016). Multivesicular body formation enhancement and exosome release during endoplasmic reticulum stress. *Biochemical and Biophysical Research Communications*.

[33] Huang H., An Y., Jiao W., Wang J., Li S., Teng X. (2018). CHOP/caspase-3 signal pathway involves in mitigative effect of selenium on lead-induced apoptosis via endoplasmic reticulum pathway in chicken testes. *Environmental Science and Pollution Research International*.

[34] Chen Z., Wen D., Wang F., Wang C., Yang L. (2019). Curcumin protects against palmitic acid-induced apoptosis via the inhibition of endoplasmic reticulum stress in testicular Leydig cells. *Reproductive Biology and Endocrinology*.

[35] Li W., Jiang Y., Sun T., Yao X., Sun X. (2016). Supplementation of procyanidins B2 attenuates photooxidation-induced apoptosis in ARPE-19 cells. *International Journal of Food Sciences and Nutrition*.

[36] Li F., Yang Y., Yang L. (2017). Resveratrol alleviates FFA and CCl4 induced apoptosis in HepG2 cells via restoring endoplasmic reticulum stress. *Oncotarget*.

[37] Chen S., Yang S., Wang M. (2020). Curcumin inhibits zearalenone-induced apoptosis and oxidative stress in Leydig cells via modulation of the PTEN/Nrf2/Bip signaling pathway. *Food and Chemical Toxicology*.

[38] Ben Salem I., Prola A., Boussabbeh M. (2015). Crocin and quercetin protect HCT116 and HEK293 cells from zearalenone-induced apoptosis by reducing endoplasmic reticulum stress. *Cell Stress & Chaperones*.

[39] Wang N., Li P., Wang M. (2018). The protective role of Bacillus velezensis A2 on the biochemical and hepatic toxicity of zearalenone in mice. *Toxins*.

[40] Chen D., Ning F., Zhang J., Tang Y., Teng X. (2020). NF-*κ*B pathway took part in the development of apoptosis mediated by miR-15a and oxidative stress via mitochondrial pathway in ammonia-treated chicken splenic lymphocytes. *Sci. Total Environ.*.

[41] Jiao W., Han Q., Xu Y., Jiang H., Xing H., Teng X. (2019). Impaired immune function and structural integrity in the gills of common carp (*Cyprinus carpio* L.) caused by chlorpyrifos exposure: through oxidative stress and apoptosis. *Fish & Shellfish Immunology*.

[42] Avery J. C., Hoffmann P. R. (2018). Selenium, selenoproteins, and immunity. *Nutrients*.

[43] Nogales F., Ojeda M. L., Fenutría M., Murillo M. L., Carreras O. (2013). Role of selenium and glutathione peroxidase on development, growth, and oxidative balance in rat offspring. *Reproduction*.

[44] Rayman M. P. (2004). The use of high-selenium yeast to raise selenium status: how does it measure up?. *The British Journal of Nutrition*.

[45] Ji Y. L., Wang Z., Wang H. (2012). Ascorbic acid protects against cadmium-induced endoplasmic reticulum stress and germ cell apoptosis in testes. *Reproductive toxicology*.

[46] Chen Q. Q., Yuan A. H., Yang J., Zha B. X., Zhang M. (2017). Effect of acupuncture on the endoplasmic reticulum stress IRE1-CHOP pathway and the expression levels of Bax and Bcl-2 protein as well as genes in pancreatic tissue of rats with diabetes mellitus. *World Journal of Acupuncture - Moxibustion*.

[47] Tao Y. K., Yu P. L., Bai Y. P., Yan S. T., Zhao S. P., Zhang G. Q. (2016). Role of PERK/eIF2*α*/CHOP endoplasmic reticulum stress pathway in oxidized low-density lipoprotein mediated induction of endothelial apoptosis. *Biomedical and Environmental Sciences*.

[48] Lam C. F., Yeung H. T., Lam Y. M., Ng R. K. (2018). Reactive oxygen species activate differentiation gene transcription of acute myeloid leukemia cells via the JNK/c-JUN signaling pathway. *Leukemia Research*.

[49] Mitomo S., Omatsu T., Tsuchiaka S., Nagai M., Furuya T., Mizutani T. (2016). Activation of c-Jun N-terminal kinase by Akabane virus is required for apoptosis. *Research in Veterinary Science*.

[50] Zhang J., Zhang Z., Bao J. (2017). Jia-Jian-Di-Huang-Yin-Zi decoction reduces apoptosis induced by both mitochondrial and endoplasmic reticulum caspase12 pathways in the mouse model of Parkinson’s disease. *Journal of Ethnopharmacology*.

